# Case report: A novel nonsense mutation in the *MARVELD2* gene causes nonsyndromic hearing loss in a China family

**DOI:** 10.3389/fgene.2024.1507600

**Published:** 2024-12-04

**Authors:** Chuican Huang, Zhenning Huang, Ping Wang, Xijing Wu, Qiaomiao Zhou, Jun Ding, Qing Luo, Weijia Wu, Xialin Fan, Lichun Fan

**Affiliations:** ^1^ Hainan Women and Children’s Medical Center, Hainan Medical University, Hainan Academy of Medical sciences, Haikou, China; ^2^ School of Basic Medicine and Life Science, Hainan Medical University, Hainan Academy of Medical Sciences, Haikou, China; ^3^ School of Pediatrics, Hainan Medical University, Hainan Academy of Medical Sciences, Haikou, China

**Keywords:** Deafness gene, MARVELD2, Nonsyndromic hearing loss, nonsense mutation, tricellulin

## Abstract

The *MARVELD2* gene is located on chromosome 5q13.2 and is associated with autosomal recessive nonsyndromic hearing loss (OMIM: # 610572). In this study, we identified and reported a novel nonsense mutation in *MARVELD2* c. 663G > A in a Chinese family. We collected peripheral venous blood from 19 members of the affected family and performed whole exome sequencing to analyze the mutation genotype. A single-nucleotide mutation was detected in *MARVELD2*. Five individuals in the family carried the *MARVELD2* c.663G>A mutation; one of them was homozygous and showed severe congenital deafness and language impairment. The next-generation sequencing results were validated by Sanger sequencing. This study expands the spectrum of *MARVELD2* mutations that cause nonsyndromic hearing loss and provides insights into the molecular pathogenesis underlying deafness. This finding has important implications for genetic screening, diagnosis, counseling, and research of deafness-related genes.

## 1 Introduction

Nonsyndromic hearing loss (NSHL, OMIM: # 610572) is a common congenital condition. Genetic factors are the major cause of NSHL, accounting for more than 70% of the cases (Wang et al., 2018; Smith et al., 2005). To date, at least 127 genes and 170 loci have been implicated in the pathogenesis of NSHL (Morgan et al., 2019; An et al., 2019). *MARVELD2*, located at the DFNB49 locus on chromosome 5q13.2, is one of the genes associated with NSHL (Sadeghi et al., 2020). Mutations in *MARVELD2* can result in bilateral, moderate to severe NSHL (Zheng et al., 2019). *MARVELD2* contains seven exons and encodes a 558 amino acid protein called tricellulin. Tricellulin is a tight junction protein that is expressed in the inner ear epithelial cells and plays a crucial role in maintaining the barrier and fence functions of the tight junctions. Tricellulin regulates the transport of water, ions, and large and small molecules using the paracellular pathway and restricts the free flow of cell membrane lipids and proteins (Gao et al., 2023; Mariano et al., 2011). It controls lateral ion diffusion that is essential for normal hearing. Therefore, *MARVELD2* mutations may cause varying degrees of hearing loss (Taghipour-Sheshdeh et al., 2019; Kitajiri and Katsuno, 2016; Kitano et al., 2019; Kamitani et al., 2015).

In this case, we evaluated a newborn male child who failed the screening test for hearing; but was found to have normal hearing upon further evaluation. However, both his parents had hearing impairments. Genetic testing of the child revealed the presence of the *MARVELD2* c.663G>A and the *MT-RNR1* 1555A>G mitochondrial gene mutations. Family tracing analysis revealed that the father of the child was the proband, with a homozygous *MARVELD2* c.663G>A mutation. The child’s *MT-RNR1* 1555A>G mitochondrial gene, encoding the 12S rRNA mutation, was inherited from the maternal lineage via an extranuclear (mitochondrial) inheritance pattern, whereas the heterozygous *MARVELD2* c.663G>A mutation was inherited from the homozygous father.

This study aimed to analyze the molecular, epidemiological, and clinical phenotypes of the members of a family with deafness carrying the *MARVELD2* c.663G>A nonsense mutation. We aimed to provide a basis for future deafness gene-related genetic screening and identification, genetic counseling and prevention of NSHL, and to provide valuable guidance for the diagnosis and research of deafness genes. Our study has enriched the mutation spectrum of *MARVELD2* and improved our understanding of its role in NSHL, which has significant implications for clinical diagnosis and management.

## 2 Case description

A 1-year-old boy failed the postnatal hearing screening test at the Hainan Provincial Women and Children’s Medical Center. After consultation, researchers found that both parents of the child were deaf, and there was no consanguineous marriage in the family. Further hearing evaluation revealed that the boy’s hearing was normal. Genetic testing revealed that he harbored a compound heterozygous mutation *MARVELD2* c.663G>A and *MT-RNR1* 1555A>G. Gene tracking of the family revealed that the father was a proband of the *MARVELD2* c.663G>A mutation. The heterozygous 12S rRNA gene mutation encoded by the *MT-RNR1* 1555A>G mitochondrial gene carried by the child was inherited from the maternal lineage, and the heterozygous *MARVELD2* c.663G>A gene mutation was inherited from the homozygous father. This study was approved by the Ethics Committee of the Hainan Women and Children’s Medical Center. All participants provided informed consent for genetic testing and release of their case details.

**FIGURE 1:**
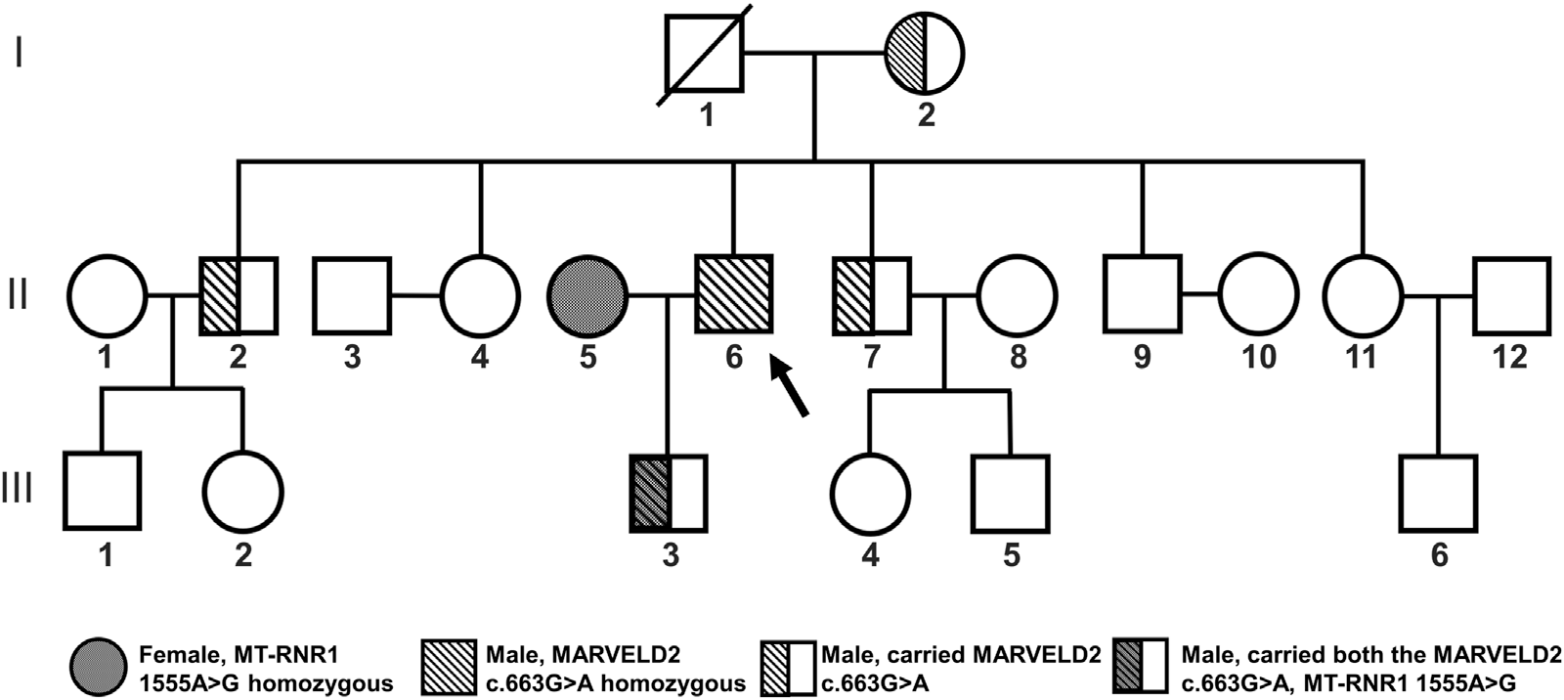
Familial pedigree of *MARVELD2* with an autosomal recessive inheritance pattern. The *MARVELD2* c.663G>A mutation and the *MT-RNR1* 1555A>G mutation are shown in the pedigree. Individual II6 is the proband of MARVELD2 c.663G>A. The grandmother and two brothers (I2, II2, II7) were carriers of the *MARVELD2* c.663G>A mutation. The grandfather (I1) passed away.

The proband (Figure 1 II6) was homozygous for the *MARVELD2* c.663G>A mutation and showed profound congenital deafness and language impairment. In this family, the grandparents had normal hearing, but the grandfather (Figure 1 I1) who had passed away, the grandmother, and two brothers (Figure 1 I2, II2, II7) were heterozygous carriers of the *MARVELD2* c.663G>A mutation although their hearing is normal.

## 3 Results

**FIGURE 2:**
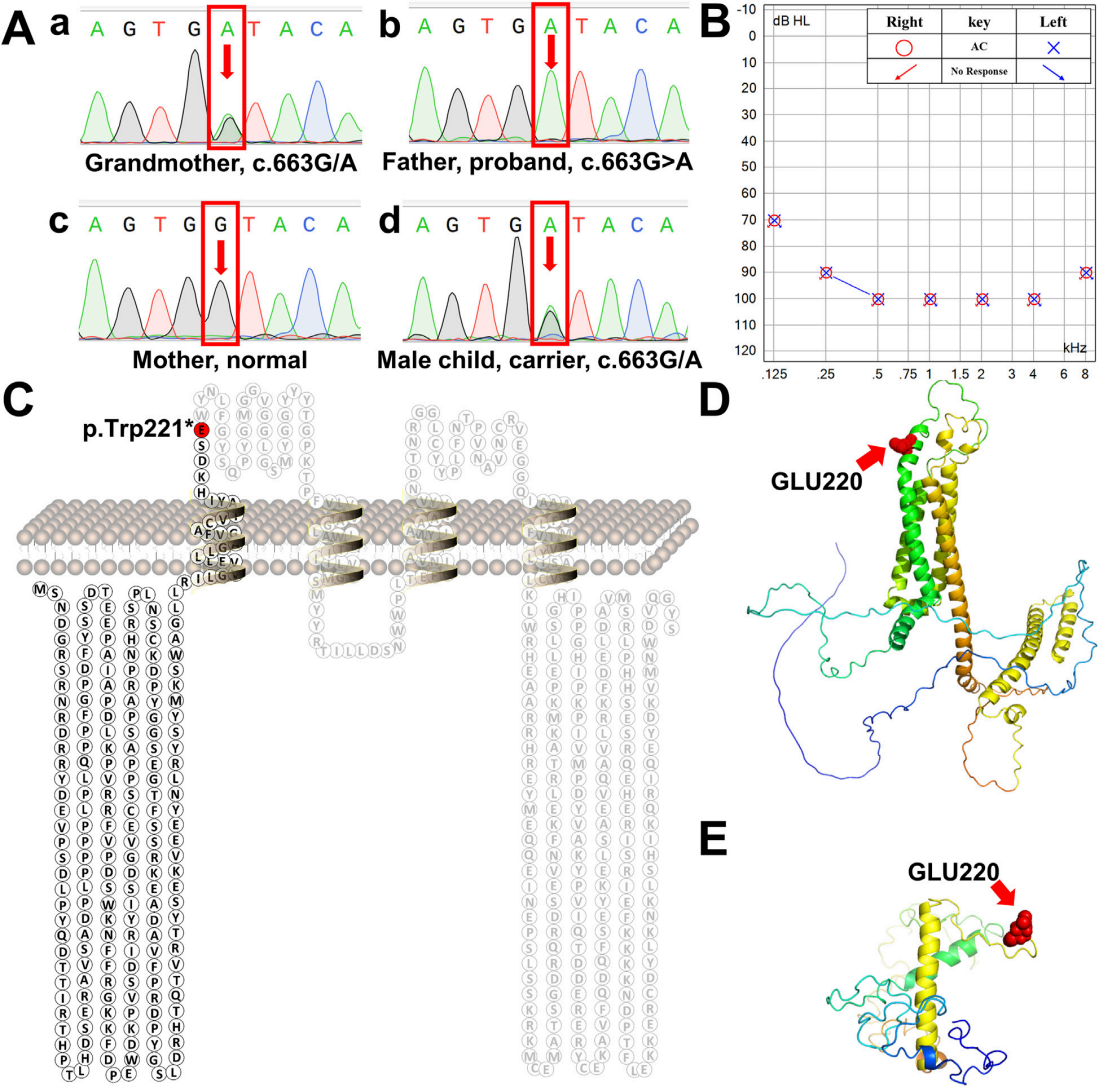
Schematic diagram of human tricellulin protein and its variant. **(A)** Sanger sequencing chromatograms of the *MARVELD2* gene confirmed a point mutation in this family. a-d are the chromatograms of a control individual, the grandmother the proband proband’s wife, and proband’s son, respectively. Arrows indicate the position of the single-nucleotide mutation. **(B)** The proband’s bilateral hearing test result showed that both ears had profound hearing loss. **(C)** Protein secondary structure of human tricellulin. The red residue marks the position of protein truncation. **(D)** 3D structure of normal human tricellulin. The red shows the position of the mutation. **(E)** 3D structure of the *MARVELD2* **(C)**663G>A mutated truncated protein. The red spheres show the position of the mutation. The 3D structure was predicted using the I-TASSER software.

We performed family pedigree analysis and next-generation sequencing (NGS) to confirm that the father (Figure 1 II6) was the proband who carried the pathogenic mutation *MARVELD2* c.663G>A. This mutation caused autosomal recessive NSHL with severe bilateral hearing loss (Figure 2B). The other four individuals (Figure 1 I2, II2, II7, III3) in the family were heterozygous carriers of the *MARVELD2* c.663G>A mutation and had normal hearing.

NGS analysis revealed the novel mutation, c.663G>A, in the *MARVELD2* gene. The Sanger verification electropherograms validated that the father was homozygous for the pathogenic mutation *MARVELD2* c.663G>A, which resulted in a nucleotide substitution of G by A at position c.663. The mother had a wild-type *MARVELD2* gene. Their son was heterozygous for the pathogenic mutation *MARVELD2* c.663G>A (Figure 2A). The raw sequence reads have been uploaded to the National Center for Biotechnology Information database (NCBI; GenBank accession no. PRJNA976897). Up to now, 12 mutations of MARVELD2 causing NSHL have been reported, as shown in Table 1. However, the MARVELD2 mutation identified in this study was not found in any of the previously reported studies.

**TABLE 1:** Summary of all reported pathogenic variants and related information in *MARVELD2*.

Variant	Amino acid substitution	Mutation type	Exon	Completeness (%)	References	Population
c.663G>A	p.W221*	Nonsense	2/7	39.61	This report	Chinese
c.730G>A	p.G244R	Missense	2/7	100.00	Zheng et al. (2019)	Chinese
c.772G>A	p.V258M	Missense	2/7	100.00	Zheng et al. (2019)	Chinese
c.949C>G	p.R317G	Missense	2/7	100.00	Zheng et al. (2019)	Chinese
c.1006C>T	p.R336W	Missense	2/7	100.00	Zheng et al. (2019)	Chinese
c.1183-1G>A	p.C395fs*396	Frameshift	4/7	70.97	Riazuddin et al. (2006)	Pakistani
c.1331 + 1G>A	-	Splice donor	4/7	-	Chishti et al. (2008)	Pakistani
c.1331 + 2T>C	p.C395fs*403	Frameshift	4/7	72.22	Riazuddin et al. (2006)	Pakistani
c.1331+2delTGAG	p.K445fs*461	Frameshift	4/7	82.61	Riazuddin et al. (2006)	Pakistani
c.1498C>T	p.R500*	Nonsense	5/7	89.61	Riazuddin et al. (2006)	Pakistani
Exon4_5deletion	p.C395-Q501del	Deletion	(4-6)/7	80.82	Nayak et al. (2015)	Pakistani
c.1543delA	p.K517Rfs*16	Frameshift	6/7	95.52	Babanejad et al. (2012)	Iranian
c.1555delinsAA	p.D519Kfs*12	Frameshift	7/7	95.16	Sarmadi et al. (2020)	Iranian

As mentioned in Section 1, *MARVELD2* c.663G>A introduces a nonsense mutation, that leads to truncated transcripts of the protein (Figure 2C) due to the change to a premature stop codon (p.Trp221*). We used three-dimensional structure model prediction (I-TASSER, S731348, and S730824), bioinformatic analysis, and database annotation to assess the pathogenicity of this mutation. We found that the location of the nonsense mutation results in the loss of essential domains of the tricellulin protein, such as the occludin-ELL domain, which is critical for the protein’s function (Mariano et al., 2011; Li et al., 2005). This caused the proband to present with severe NSHL (Figures 2B, E, F). These domains are involved in protein-protein interactions and tight junction formation, which are important for normal hearing.

## 4 Discussion

NSHL is a common sensorineural disorder with an increasing incidence. NSHL exhibits genetic heterogeneity, with most cases showing autosomal recessive inheritance (Safka Brozkova et al., 2012). *MARVELD2* encodes a four-transmembrane protein consisting of seven exons and 558 amino acids (Riazuddin et al., 2006; Nayak et al., 2013). Mutations in human *MARVELD2* which is located at the DFNB49 locus have been reported to cause NSHL in different studies (Nayak et al., 2015). In this study, we used NGS and Sanger sequencing to evaluate a family with NSHL, identifying a rare mutation (c.663G>A) in *MARVELD2*. The mutation caused the tricellulin protein to become truncated at glutamic acid position 221, resulting in a 60.4% amino acid loss compared to the wild-type protein. To the best of our knowledge, this is the first report of the identification of the mutation.

Tricellulin is ubiquitously expressed in epithelial junctions of tissues and organs throughout the body. However, the only obvious phenotype of the *MARVELD2* mutant alleles is deafness. Tricellulin encoded by *MARVELD2* contains two conserved domains with important functions, four-transmembrane domain and an Occludin-ELL domain (Pfam accession number PF07303) located at the C-terminus. The tricellulin C-terminus interacts with the cytosolic adapter protein ZONA occludens-1 (ZO-1) and is thereby linked to the actin cytoskeleton (Riazuddin et al., 2006; Ikenouchi et al., 2005). Mutations that cause structural damage to the C-terminus in patients with NSHL result in restricted binding to ZO-1. This observation emphasizes the importance of the C-terminal domain for the physiological function of tricellulin. For example, an affected NSHL family member in a Pakistani family was homozygous for a transition mutation (c.1498C>T) that creates a nonsense codon p.R500X in exon five of *MARVELD2*, which results in a truncation within the occludin-ELL domain of the protein’s C-terminus (Riazuddin et al., 2006). In another four cases in Pakistani family (c.1183-1G>A, c.1331 + 1G>A, c.1331 + 2T>C, c.1331+2deITGAG), mutations occurred at the splice donor where exon 4 and intron 4 intersect, changing the original splicing position of hnRNA and leading to premature termination of protein translation (Riazuddin et al., 2006; Chishti et al., 2008; Nayak et al., 2015). In addition, two variants c.1555delinsAA and c.1543delA occurred in Iranian families, resulting in frameshift mutations that caused premature stop codons (Babanejad et al., 2012; Sarmadi et al., 2020). These variants all resulted in the lack of complete domains of the terminal occludin-ELL encoded by exons 5, 6, and 7. All reported variants that affect the integrity of the occludin-ELL domain have led to the occurrence of NSHL. The new truncation mutation identified in our study results in direct deletion of the C-terminus, causing severe hearing impairment.

The proband was homozygous for the *MARVELD2* mutation due to its recessive inheritance pattern, and there was no consanguineous marriage within the family, which originated from Hainan Province, China. Because the mutation shows recessive inheritance, it indicates that the mutated gene has a certain carrying scale in Hainan Province. No large-scale epidemiological surveys have been conducted to estimate the prevalence of this mutation. Further research is required to determine its incidence and carrier frequency. Our findings suggest that *MARVELD2* mutations must be considered in patients with NSHL in Hainan Province. Further, our study expands the spectrum of clinical manifestations associated with gain-of-function mutations in the tricellulin protein, and contributes to the identification of additional *MARVELD2* gene variants.

## 5 Conclusion

NGS and Sanger sequencing, combined with database annotation, confirmed that the nonsense mutation c.663G>A in *MARVELD2* is pathogenic and causes NSHL, and shows an autosomal recessive inheritance pattern.

## 6 Materials and methods

### 6.1 Clinical diagnosis and medical history collection

A multidisciplinary team consisting of genetic counselors and doctors from the pediatric, ENT (ear, nose and throat), and prenatal diagnosis departments visited the patient’s family. The patient’s medical history was collected and a specialist examination was conducted by an experienced ENT doctor. Details of the medical history that were collected included the chief complaint, general illness, present illness history, past illness history (with or without other diseases), personal history, family history, and other high-risk factors closely related to deafness.

### 6.2 DNA extraction

DNA was extracted from blood samples from each individual using a standard phenol-chloroform procedure (Grimberg et al., 1989), and the target gene was amplified using polymerase chain reaction (PCR). The samples were sent to the Shenzhen BGI Medical Testing Center (Yantian District, Shenzhen, China). After the quality and quantity of each sample was confirmed, NGS was performed for genetic testing for hereditary deafness.

### 6.3 Target sequence capture and sequencing

A minimum of 5 mL of venous blood was drawn from participants and controls, and genomic DNA was extracted using as per manufacturer’s instructions (MagPure Buffy Coat DNA Midi KF Kit, MAGEN, D3537-02). The genomic DNA was enzymatically fragmented to 200–300 bp, and the fragments were selected and end-repaired with “A”. Adapters containing labeled sequences were added to both ends of the DNA using a ligase, and a pre-PCR library was constructed via PCR amplification. Target DNA fragments in the library were hybridized with probes containing the target gene labeled with biotin (Roche, 9,062,629,001) and anchored onto streptomycin magnetic beads through a biotin-avidin reaction. After washing to remove the non-target DNA, the specifically captured enriched DNA was amplified via PCR and purified to obtain a post-PCR library. The size and concentration of the post-PCR library were determined using Caliper GX and BMG, respectively. After quality control, the library was mixed for pooling, single-stranded separation, cyclization, and rolling circle replication to generate DNA nanoballs, which were sequenced using a MGISEQ-2000 gene sequencer.

### 6.4 Sequence linkage analysis

After filtering, alignment, deduplication, and quality control of the data, mutation analysis was performed to obtain the mutation analysis results (VCF files). The mutation results were annotated via combination of the frequency and disease databases of related genes, and the impact of mutations on the protein structure was predicted to obtain the sample mutation and classification results (Ahmed et al., 2001).

After the data were downloaded, information analysis was conducted. First, the original data (raw reads) were evaluated for sequencing quality and low-quality reads contaminated by adapters were removed. Burrows Wheeler Aligner software was used to align with HG19 and the sequence capture effect was evaluated. GATK UnifiedGenotyper and GATK HaplotypeCaller software were used to query single nucleotide mutations (SNVs) and indels (insertions and deletions), respectively, to generate target region base polymorphism results. Subsequently, the databases (NCBI dbSNP, HapMap, ESP6500, ExAC, 1,000 human genome datasets, and HGMD) were aligned, and suspected mutations were annotated and screened (Richards et al., 2015; den Dunnen et al., 2016).

### 6.5 Sanger sequencing data validation

For the discovered SNV-positive mutation (c.663G>A), primer pairs were designed upstream and downstream of their fragments as follows: 5ʹ-AAA​GAG​GCT​GAC​GCA​GTG​TT-3ʹ as a forward primer and 5ʹ-TAT​GTC​TCC​GAG​CTG​CCT​CA-3ʹ as a reverse primer. PCR amplification was performed, and the PCR product was subjected to Sanger sequencing. The obtained sequence was aligned with the reference gene standard sequence to obtain mutational information, thereby verifying the results of gene chip capture and high-throughput sequencing (Tabatabaiefar et al., 2011).

## Data Availability

The datasets presented in this study can be found in online repositories. The names of the repository/repositories and accession number(s) can be found below: https://www.ncbi.nlm.nih.gov/, PRJNA976897.
